# Cost-effectiveness of personalised telehealth intervention for chronic disease management: A pilot randomised controlled trial

**DOI:** 10.1371/journal.pone.0286533

**Published:** 2023-06-15

**Authors:** Shalika Bohingamu Mudiyanselage, Jo Stevens, Julian Toscano, Mark A. Kotowicz, Christopher L. Steinfort, Robyn Hayles, Jennifer J. Watts

**Affiliations:** 1 Deakin Health Economics, Institute for Health Transformation, School of Health and Social Development, Deakin University, Geelong, Victoria, Australia; 2 Barwon Health, University Hospital Geelong, Geelong, VIC, Australia; 3 Deakin University School of Medicine, Geelong, VIC, Australia; 4 Melbourne Clinical School-Western Campus, Department of Medicine, The University of Melbourne, St Albans, VIC, Australia; National Center for Global Health and Medicine, JAPAN

## Abstract

**Objective:**

The study aims to assess the cost-effectiveness of a personalised telehealth intervention to manage chronic disease in the long run.

**Method:**

The Personalised Health Care (PHC) pilot study was a randomised trial with an economic evaluation alongside over 12 months. From a health service perspective, the primary analysis compared the costs and effectiveness of PHC telehealth monitoring with usual care. An incremental cost-effectiveness ratio was calculated based on costs and health-related quality of life. The PHC intervention was implemented in the Barwon Health region, Geelong, Australia, for patients with a diagnosis of COPD and/or diabetes who had a high likelihood of hospital readmission over 12 months.

**Results:**

When compared to usual care at 12 months, the PHC intervention cost AUD$714 extra per patient (95%CI -4879; 6308) with a significant improvement of 0.09 in health-related quality of life (95%CI: 0.05; 0.14). The probability of PHC being cost-effective by 12 months was close to 65%, at willingness to pay a threshold of AUD$50,000 per quality-adjusted life year.

**Conclusion:**

Benefits of PHC to patients and the health system at 12 months translated to a gain in quality-adjusted life years with a non-significant cost difference between the intervention and control groups. Given the relatively high set-up costs of the PHC intervention, the program may need to be offered to a larger population to achieve cost-effectiveness. Long-term follow-up is required to assess the real health and economic benefits over time.

## Introduction

Chronic disease prevalence and associated costs are a burden for many health systems [1]. A major component of healthcare expenditure is the cost of medication, diagnostics, treatments and hospitalisation. There is also a significant social burden in productivity loss, informal care and loss of Quality-Adjusted Life Years (QALYs) [2–4].

The social and economic burden from chronic diseases such as diabetes, cardiovascular disease and chronic respiratory disease is high and growing [5, 6]. Health care management of chronic disease has traditionally been focused in the hospital setting for diagnosis and acute management. However, the growing challenges and high costs of managing chronic disease have shifted care focus from the acute setting to the primary healthcare setting [7]. Previous evidence has highlighted that many chronic diseases can be treated and managed in the primary healthcare setting, supported by hospital admission for complex treatments [8].

Recently, there has been a rapid growth in telehealth technologies to diagnose, prevent, and manage chronic disease in an out-of-hospital environment. There is evidence that these new technologies have increased patients’ Quality Of Life (QOL) [9] and reduced unnecessary hospitalisations [10]. For chronic diseases such as Chronic Obstructive Pulmonary Disease (COPD), where patients experience acute exacerbations as the disease progresses, and diabetes, where disease duration is related to complications, hospital admissions become more frequent. In an out-of-hospital supported environment, patients can be encouraged to self-manage their disease, so deterioration is detected and managed outside the hospital environment [11].

Telehealth has been advocated in managing a range of chronic conditions to reduce hospital admissions, improve self-care and improve QOL [12]. Telehealth allows sharing medical information and communication, therefore, improving access to health care between patients and clinicians regardless of geographic separation [13, 14]. Telehealth studies have shown benefits to individuals and the health system from decreased hospitalisations and emergency room visits, reduced nursing home admissions, reduced burden on health care professionals and patient transport costs [15]. A recent systematic review concluded that telehealth implementation should be focused on benefits rather than costs, as current evidence suggests that telehealth is unlikely to reduce the cost of health care delivery, but it may improve patient outcomes [15]. Research indicates that telehealth may have a positive impact on patients with chronic conditions through improved disease management, clinical indicators, QOL and health care support [16]. Telehealth provides an opportunity for patients to partner in their disease management consistent with expert patient literature [17] where, by living with and managing their condition, patients develop the ability to make informed decisions about their care [18].

A challenge to the successful implementation of telehealth interventions is identifying the patient population likely to benefit most in terms of a reduction in hospitalisations and/or an improvement in health outcomes. Cost-effectiveness is more likely to be demonstrated in a patient population that is at high risk of hospital readmission in a subsequent period.

Barwon Health Personalised Health Care (PHC) intervention is a telehealth remote patient monitoring program that aims to improve the capacity of people with chronic diseases to manage their diseases at home [19]. Through offering the PHC program, it was predicted that savings in costs of hospitalisation were likely to be achieved. The trial was undertaken at Barwon Health between 2014–15 [19]. The target population, based on the probability of readmission, was identified from patient records using Pattern Recognition and Data Analytics (PRaDA) [20]. Economic analysis compared the total cost of care and the difference in QOL outcomes. The aim of the study was to assess the cost-effectiveness of Barwon Health Personalised Health Care intervention to manage chronic disease.

## Method

This pilot randomised controlled trial with an economic analysis conducted alongside compared the PHC intervention against usual care [19]. The economic analysis was a cost-effectiveness analysis, comparing the costs of the intervention and hospitalisation between the study groups from the perspective of the health service, Barwon Health The cost-effectiveness analysis was undertaken to determine whether the intervention was cost-effective compared to usual care based on the change in health-related QOL using the Assessment of Quality of Life Eight Dimension (AQoL-8D) utility instrument [21]. The Human Research Ethics Committees Barwon Health (HREC 13/14) and Deakin University (HREC 2015–026) approved ethics for the study. The trial was registered on the Australian New Zealand Clinical Trials Registry (ACTRN12617000396325).

Patients diagnosed with COPD and/or diabetes from the Barwon Health region were eligible for the study if they had a high likelihood of hospital readmission (predicted by PRaDA) over the next 12 months [19]. Full intervention details are published elsewhere [19].

Patients randomised to the intervention were provided with clinical monitoring equipment depending on diagnosis (blood glucose monitors, blood pressure monitors and/or pulse oximeters), a tablet device with internet connection [19] and TELUS package (telehealth home monitoring software) [22].

Participants randomised to the control group received usual care. In both study groups, patients were advised that if their condition deteriorated or they experienced an acute exacerbation, they should seek medical advice as usual (via Emergency Department (ED) or General Practitioner).

The intervention costs and hospital admission data pertaining to study participants were collected from the study team and hospital costing team.

All data were re-organised according to an economic evaluation plan and transferred to Stata®13, Stata®14 and SPSS®24 format for cleaning and analysis. T-tests and 95% Confidence Intervals (CI) were used for the comparison of mean total cost, length of stay (LOS) and QALYs between the study groups.

### Economic evaluation

The data sources and assumptions used in the economic evaluation are shown in Table 1. All costs are reported as 2014 Australian dollars (AUD) to match with the main study outcome paper [19] and to keep consistency for readers. The economic evaluation was conducted from the health service perspective, which was the Barwon Health perspective. The costs to Barwon Health relating to this intervention were hospitalisation costs (which include admission, treatment and pharmaceutical costs) and the intervention cost.

**Table 1:** Unit costs, assumptions & sources.

	Unit cost 2014 $	Unit	Assumptions	Source
Software licence agreement	480.00	Per licence	Costs have been attributed based on study population	Study team
Protocol licence fee	15,000.00	per year $5,000	Depreciated over 3 years	Study team
Video conferencing	15,200.00	per year $5,067	Depreciated over 3 years	Study team
Project management from suppliers	50,000.00	per year $16,667	Depreciated over 3 years	Study team
Grade 3B Y2 Community Health Nurse	44.97* 77.61**	Per hour including on costs 18%	-	Fair Work Australia [23]
Community nursing costs (on-call)	53.00	Per hour	-	Study team
Consultant Registrar	130.00	Per patient, Per hour	-	Study team
PHC Manager Grade 5 ADON	121,496.00	Per year	0.5EFT***	Study team
Staff Training	16,000.00	Cost per year $5,333	Depreciated over 3 years	Study team
Travel	0.80	Per kilometre	-	Study team
Pulse Oximeter	523.00	Per unit	Depreciated over 3 years	Study team
Blood Glucose Monitor	75.00	Per unit	Depreciated over 3 years	Study team
Blood Pressure Monitor	190.00	Per unit	Depreciated over 3 years	Study team
Tablet with a case	885.00	Per unit	Depreciated over 3 years	Study team
Internet connection	504.00	Per participant	Depreciated over 3 years	Study team
Installation Fees	50.00	Per unit	Depreciated over 3 years	Study team
Average cost of hospital bed (modelled)	-	Per day	Costs will depend on individual DRG**** and base weight	Victoria [24] Government Health

*hourly rate of weekday

** average hourly rate for weekend

*** Fulltime work (35 hours)

****Diagnosis Related Group

### Intervention costs

Fixed costs related to the software licence agreement, protocol licence, project management and video conferencing were provided by the study team. All fixed costs were depreciated over 3 years on the assumption that the program would be viable for 3 years before an upgrade might be necessary.

The equipment price list was provided by the study team. All equipment was assumed to have a life of 3 years.

The mean labour cost per participant was calculated using service data provided by the study team. A nurse unit manager and two dedicated clinical nurses were employed full-time seven days per week. If the patient required further clinical attention, they were referred to their general practitioner, or at times the consultant at the hospital was available to support complex needs. Total labour cost was calculated using staff time on intervention plus additional staff training for the intervention. The total travel cost was based on the average cost of running a car and distance. In the study, the nursing staff made three visits to patients in the intervention group and two visits to patients in the control group.

### Hospitalisation-related admissions and costs

All acute and sub-acute admissions to Barwon Health in the 12-month period were included in the cost analysis for both study groups. Non-admitted patient services and same-day cancer treatment episodes were excluded. Private hospital admissions were self-reported by the patient, and with consent, private hospitals released Diagnosis Related Group (DRG) and hospital LOS information to the research team. Costs attributed to the private hospital admission and non-Barwon Health subacute admissions were modelled from Barwon Health admission records using the patient’s hospital LOS, DRG, admission type and whether the admission was public or private. National hospital cost weights [25] for admissions were derived from the Victorian Department of Health’s Weighted Inlier Equivalent Separation calculator [26] for acute and sub-acute inpatients in Victorian hospitals in 2014–15.

### Total cost

The total cost of the intervention and hospitalisation over the 12-month study period were analysed for each individual. The mean total cost per participant was compared between study groups.

### Quality of Life

**Fig 1:**
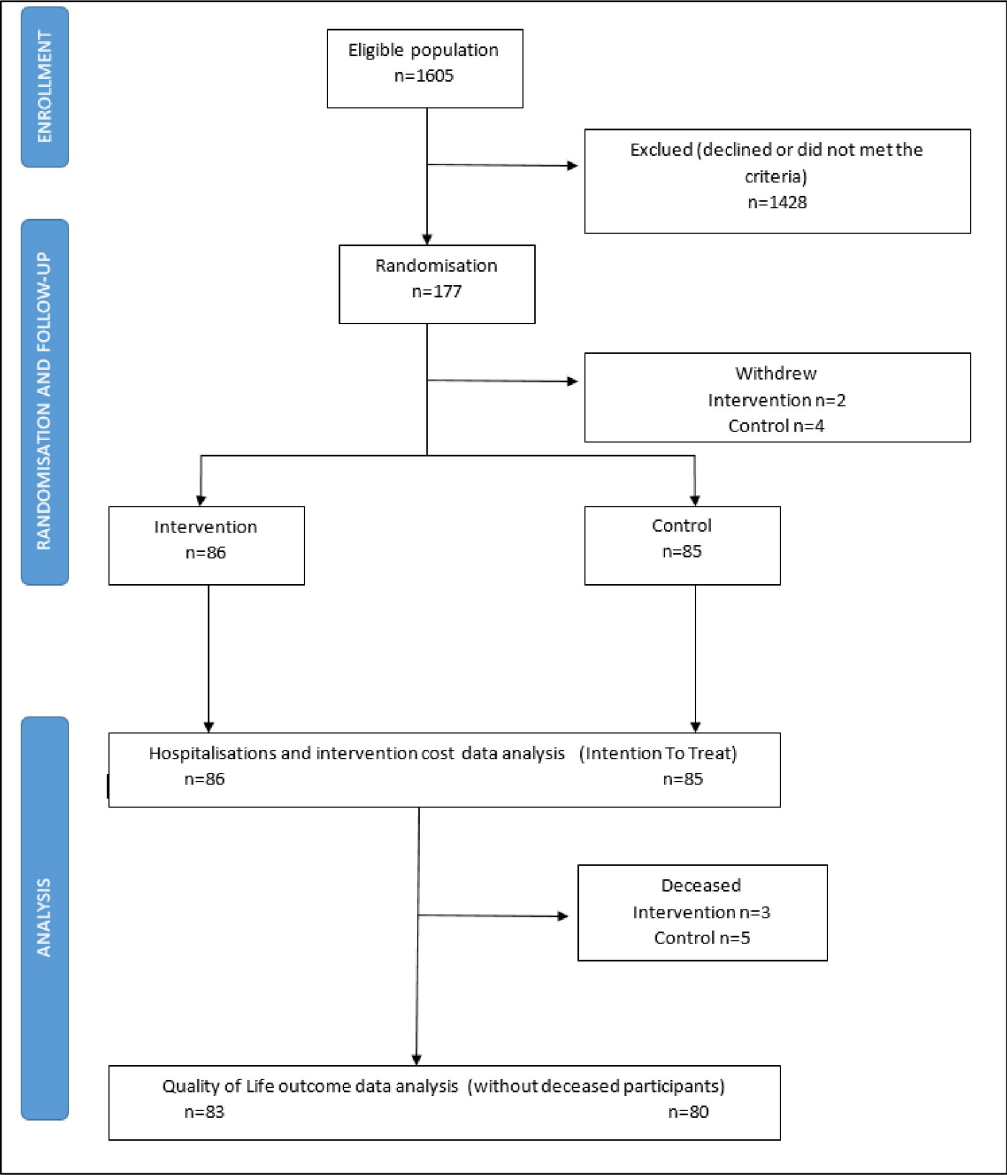
Consort chart.

The AQOL-8D instrument comprised of 35 items, derived from 8 dimensions (independent living, pain, senses, mental health, happiness, coping relationships and self-worth) and 2 super dimensions (physical and psycho-social) [21]. AQoL-8D attributes were converted to a health-related utility score based on Australian weights provided with the AQoL-8D to derive an overall utility-based HRQOL measure [21, 27, 28]. QOL data were analysed, excluding any mortalities (Fig 1). The mean difference in the utility score between the two-time points for each individual in the PHC group was calculated as final minus baseline, and the mean difference was determined between study groups. The assumption was that a utility score of 1 was equivalent to one year in full health, representing one QALY saved. A straight line relationship in the change in the utility score over 12 months was assumed.

Intention-to-treat analysis was undertaken based on the initial randomised population for costs, including hospital costs. QOL data have been analysed based on the starting population for whom AQoL-8D baseline data were recorded.

The ICER is calculated as the difference in cost between study groups divided by the difference in QALY outcomes [29]. A cost-effectiveness plane showing 95% CI around the ICER was generated using the bootstrap method (5000 simulations) and a cost-effectiveness acceptability curve showing the probability of cost-effectiveness calculated assuming different willingness-to-pay values, including the Australian threshold $50,000 per QALY [30, 31].

Modelled analyses were undertaken to account for the originally planned population of 200 participants in the intervention. We modelled the spread of fixed intervention and staffing costs over the assumption of 200 participants instead of the actual 86 participants (Fig 1).

## Results

Eighty-five patients were randomised to the control group, and 86 participants were randomised to the intervention group (Table 2). The mean age for the study population was 71 years. The mean PRaDA score, which measured the probability of readmission, was slightly higher in the intervention group 0.56 (SD 0.18) compared to the 0.50 (SD 0.21) control group (P = 0.033) (Table 2).

**Table 2:** Baseline population demographics.

	PHC Intervention	Usual care	P value
**Number of participants (n, %)**	86 (50%)	85 (50%)	-
**Gender**			
Male (n, %)	49 (55%)	40 (45%)	-
Female (n, %)	37 (46%)	45 (54%)	-
**Mean Age (years)**	70.7 (SD 11.56)	70.1 (SD 13.26)	0.383
**Diagnosis**			
Diabetes (n, %)	59 (49%)	62 (48%)	-
COPD (n, %)	19 (54%)	16 (46%)	-
Both (n, %)	8 (53%)	7 (47%)	-
PRaDA Score (mean, SD*)	0.56 (SD 0.18)	0.50 (SD 0.21)	0.033
**AQoL-8D Score (mean, SD)**	0.58 (SD 0.22)	0.58 (SD 0.21)	0.872

*Standard Deviation

### Economic evaluation

#### Intervention costs

The total fixed cost of the intervention for the study population was $68,026, and the mean fixed cost for the PHC technology per participant in the intervention group was $791. The mean variable cost per participant in the intervention group related to the delivery of the PHC intervention was $6,560 (Table 3).

**Table 3:** Variable costs.

PHC Variable costs	Costs per participant per annum 2014 $
Mean labour cost	5,583
Mean travel cost*	21
Mean equipment cost	955
**Mean total variable cost**	**6,560**

*Total travel miles for the population (2,277 km)

#### Hospitalisation-related admissions and costs

The number of admissions, LOS, and mean cost of hospitalisation in the study groups are reported in Table 4. There were 232 admissions during the study period (intervention group n = 102 and control group n = 130). The mean number of hospital admissions for the intervention group was 1.19 (SD 1.56) compared to 1.53 (SD 2.00) for the control group. There was a statistically significant difference in the mean acute hospital LOS over 12 months, for the intervention group 4.56 (SD 11.71) days compared to the control group 8.66 (SD 17.35) days (MD 4.10; (95%CI: -8.56;-0.36); P = 0.036).

**Table 4:** Hospitalisation-related admissions and costs over 12 months.

	PHC Intervention n = 86	Usual care n = 85	Mean difference (95%CI)	p-value
**Hospital admissions**				
Total number of hospital admissions (n)	102	130	-28	-
Acute admissions	97	125	-28	-
Public Hospital (acute + subacute)	96	119	-23	-
Private Hospital	1	6	-5	-
Subacute admissions	5	5	0	-
**Mean number of hospital admissions**	**1.19(SD 1.56)**	**1.53(SD 2.00)**	**-0.34(-0.19; 0.88)**	**0.106**
Acute admissions	1.13(SD 1.48)	1.47(SD 1.91)	-0.34(-0.17;0.85)	0.095
Subacute admissions	0.06(SD 0.24)	0.06(SD 0.24)	0 .01(-0.07; 0.07)	0.493
**Hospital Length of Stay**				
Total hospital days	489	814	-325	-
Acute admissions	392	736	-344	-
Public Hospital	386	709	-323	-
Private Hospital	6	27	-21	-
Subacute admissions	97	78	19	-
**Mean hospital length of stay**	**5.69(SD 16.36)**	**9.58(SD 19.97)**	**- 3.89(-9.40;1.62)**	**0.083**
Acute admissions	4.56(SD 11.71)	8.66(SD 17.35)	- 4.10(-8.56;-0.36)	0.036
Subacute admissions	1.13(SD 5.20)	0.92(SD 4.02)	- 0.21(-1.61;1.19)	0.616
**Hospital Costs**				
Acute admissions (public and private) ($)	4,651 (SD 10,085)	11,271 (SD 20,705)	-6,620(-11,528;-1,712)	0.004
Public Hospital only ($)	4,504 (SD 9,473)	11,009 (SD 20,638)	-6505(-11,344;-1,666)	0.004
Subacute admissions($)	860 (SD 4,219)	792 (SD 33,75)	67.18(-12,21;1,087)	0.454
**Mean hospital costs ($)**	**5,510 (SD 13,508)**	**12,063 (SD 22,482)**	**-6,553(-12,145;-961)**	**0.011**

**Fig 2:**
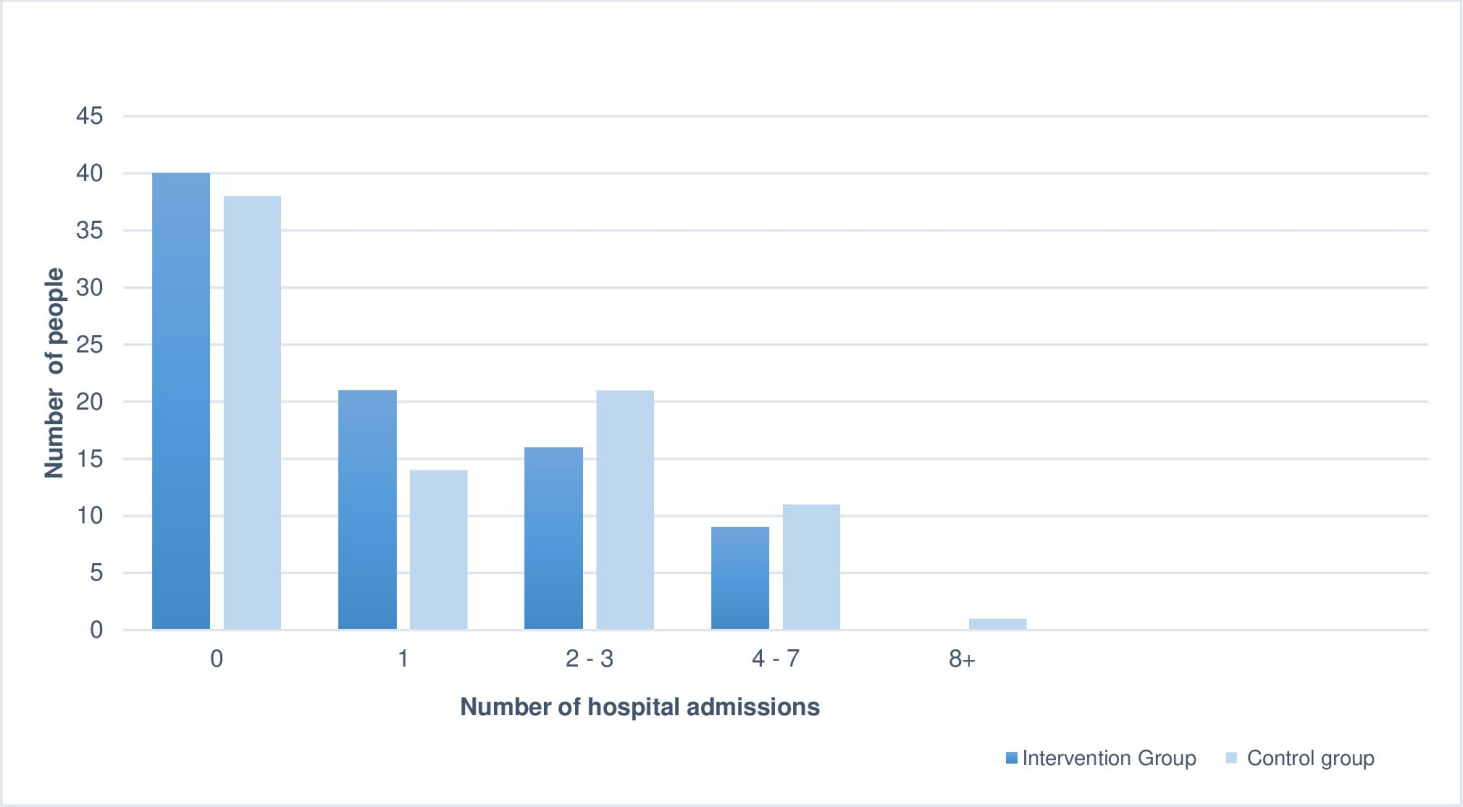
Hospital admissions per person over 12 months.

Fig 2 shows the number of admissions per person over 12 months separately for study groups. There were more people in the intervention with no hospital admission (n = 40) than in the control group (n = 37). There were also more people in the intervention group (n = 21) that had only one admission compared to the control group (n = 14). For people with multiple admissions, there were 33 patients in the control group with two or more admissions compared to 25 patients in the intervention group.

#### Total cost

Mean total costs, including intervention and hospitalisation costs, were $12,796 for the intervention group and $12,081 for the control group, with a mean cost difference of $714 (95%CI: -4879, 6308), which was favourable to the control group but not statistically significant (Table 5).

**Table 5:** Mean total cost and cost difference of Personalised Health Care intervention vs usual care.

	PHC Intervention n = 86	Usual care n = 85	Mean difference (95%CI)
**Mean fixed costs ($)**	**791**	**0**	**791**
**Mean variable costs ($)**	**6,560 (SD 74)**	**17 (SD 11)**	**6,541 (6,525;6,558)**
Mean equipment costs	955 (SD 74)	17 (SD 11)	937 (921;-953)
Mean labour costs	5,584	0	5,584
Mean travel costs	21	0	21
**Mean hospital costs ($)**	**5,510 (SD 13,508)**	**12,063 (SD 22,482)**	**-6,553 (-12,145; -961)**
Acute admissions (public and private)	4,651 (SD 10,085)	11,271 (SD 20,705)	-6,620 (-11,528;-1,712)
Subacute admissions	860 (SD 4,219)	792 (SD 3,375)	67.18 (1,221;1,087)
**Mean total cost**	**12,796 (SD 13,528)**	**12,081 (SD 22,479)**	**714 (-4,879;6,308)**

#### Quality of life

There was no difference in the mean AQoL-8D utility score between study groups at baseline. At 12 months, the QOL utility score in the intervention group had increased by 0.05. However, in the control group, it had decreased by 0.04. The difference of 0.09 (0.05; 0.14) over 12 months between the two groups was statistically significant in favour of the intervention group (Table 6).

**Table 6:** Health-related quality of life.

Outcome	Baseline		12 months		Mean difference (95%CI)
	Intervention n = 86	Usual care n = 85	Intervention n = 83	Usual care n = 80	(Intervention–Usual care)
**AQoL-8D score**	0.58 (SD 0.22)	0.58 (SD 0.21)	0.63 (SD 0.03)	0.54 (SD 0.03)	0.09 (0.05;0.14)

**Fig 3:**
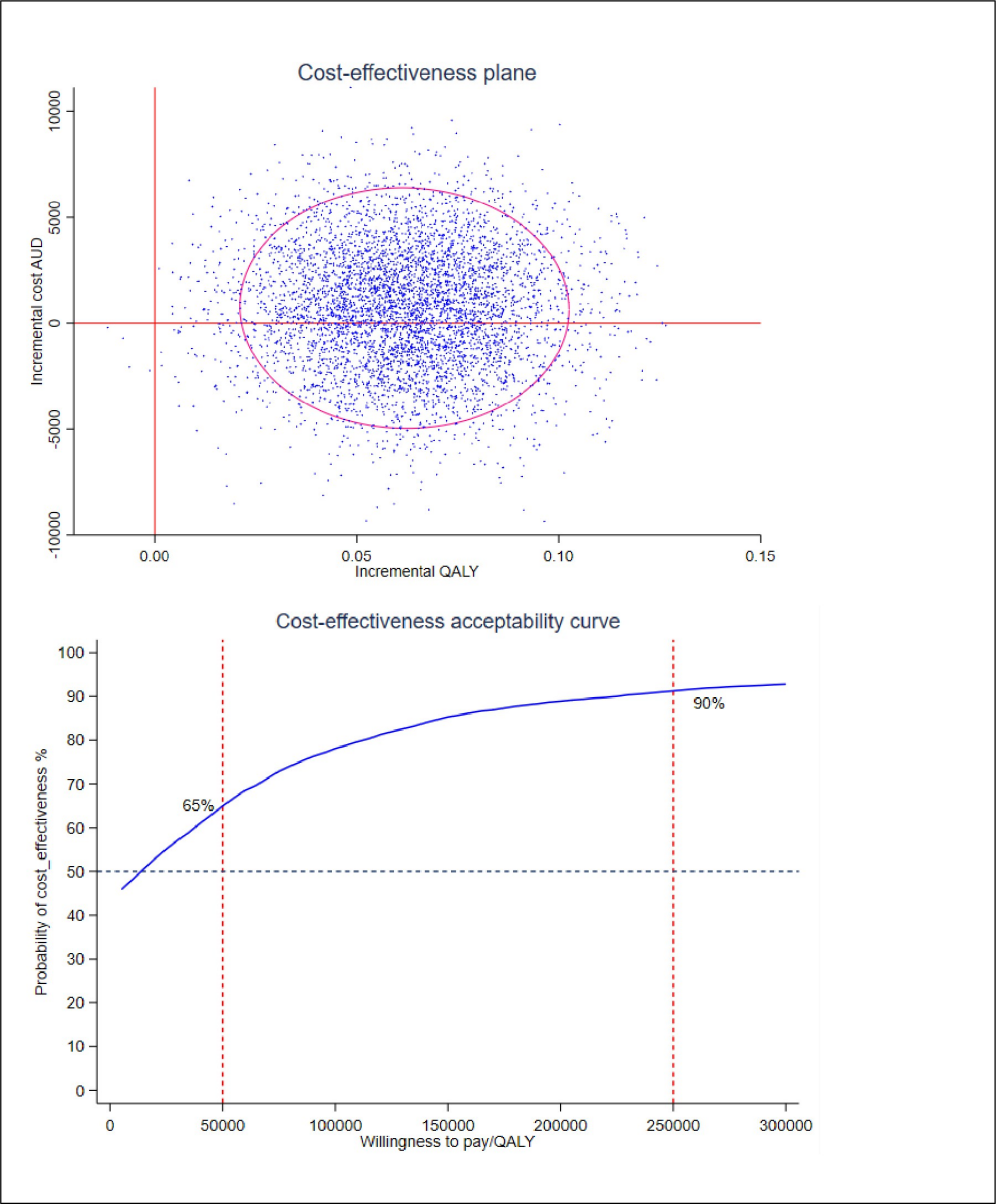
Cost-effectiveness plane and cost-effectiveness acceptability curve.

The ICER based on the incremental cost per QALY saved between study groups was a cost of $7,933 per QALY saved. The cost-effectiveness plane showed that for all simulated cases within the 95% confidence ellipse, there was a QALY improvement between study groups (cases are in both the upper and lower right quadrants). However, slightly more than half the simulated cases within the 95% confidence ellipse were in the upper right quadrant, suggesting that for these cases, the costs were higher in the intervention group compared to the control group. The cost-effectiveness acceptability curve shows that the probability of cost-effectiveness at a willingness-to-pay of $50,000 per QALY is 65%, and at $250K per QALY, the probability is 90% (Fig 3).

The modelled analysis indicated that the mean cost difference between study groups would have been reduced if the fixed costs of the intervention had been attributed to the original target population of 200 people. Savings in total cost was calculated as $2,532 (95%CI -8126; 3061); though not statistically different, the total cost was lower in the intervention group. The modelled analysis based on the variation of fixed costs over 200 people, the ICER was calculated as a savings of $28,189 per QALY, favouring the intervention group.

## Discussion

Participants in the intervention group experienced fewer hospital admissions with a reduced LOS over 12 months compared to the control group, contributing to overall savings in the hospitalisation of $6,550 per person. This result was statistically significant, and based on this result, the savings to Barwon Health from hospital costs alone would have been $550K in 2014/15. However, the savings in hospitalisation did not offset the costs of the intervention, with the overall result being a non-significant difference in the mean total cost of $714 per person in favour of the control group. In 2014 a similar Danish study for COPD patients over 12 months showed a statistically significant mean cost of $1880 (95%CI:-1447;5207) between study groups [32].

The intervention resulted in a saving of 344 acute hospital bed days over 12 months. The average LOS for overnight separations in Australian public and private hospitals was 5.5 days in 2015/16 [33], equating to 63 additional patients that could be admitted.

The mean change in QOL between groups was both statistically and clinically significant and above the minimally important difference of 0.06 [34]. A difference of 0.09 QALYs favouring the intervention group is equivalent to an extra month in full health per person. The statistically significant change in HRQOL is surprising given the small sample size, and the sample not powered to detect the difference in QOL. The PHC telemonitoring was managed by Barwon Health, a regional health service with remote monitoring and feedback coordinated by hospital staff. This may have provided an additional level of reassurance that was reflected in the HRQOL scores. The difference in hospital admissions may also have impacted HRQOL. A systematic review of telehealth interventions for asthma also found a significant difference in patient QOL [35].

The cost-effectiveness analysis showed an ICER of $7,993 per QALY saved. This means that the cost of a gain of one additional year in full health would be $7,993. While there is no publicly stated willingness-to-pay threshold for a QALY in Australia, $50,000 per QALY [30, 31] is considered acceptable in evaluating economic evidence. The ICER of $7,993/QALY for PHC compared to usual care is below the willingness-to-pay threshold of $50,000/QALY, suggesting that the PHC cost-effective at a willingness-to-pay threshold of $50,000 per QALY. Other studies that have concluded that telehealth programs are not cost-effective compared to regular care [32, 36, 37] have not taken the value of a QALY into consideration.

Modelled analysis suggested that increasing the scale of the intervention would result in a savings of $28,189 per QALY. The high fixed costs of the intervention and efficiency savings in staffing spread over an increased number of patients were accounted for in the modelled analysis where the original target population was assumed. Under this assumption, the savings in hospitalisation would offset the fixed costs of the intervention and would have resulted in an average savings of $2,532 per person.

The major strengths of this economic analysis are the inclusion of the actual intervention costs and staffing using a bottom-up costing approach to determine the real staff time to deliver the program. In addition, access to patient-level clinical costing data from Barwon Health enabled the cost of hospitalisation to be included in the analysis rather than a modelled costs based on DRG pricing for Victoria.

One limitation to the evaluation was the small study population compared to the initially planned population. Although we have tried to make assumptions for this in the modelled analysis, we cannot be sure that the assumptions concerning staffing levels are accurate. It is possible that staffing levels would need to increase to allow for increased patient numbers. Nevertheless, the mean cost per person could be reduced if the capital costs of the intervention were attributed to a larger population.

Most patients with chronic diseases have their own monitoring devices. Equipment cost was an out-of-pocket cost for the control group. However, the cost of equipment such as blood glucose monitors is unlikely to be totally out-of-pocket because some of these costs are covered under the National Diabetes Services Scheme [38]. The mean equipment cost calculated for this evaluation may represent an overestimation from the health service’s perspective.

A limitation in costing hospital episodes was not being able to cost admissions to private hospitals due to lack of access to private administrative data. If we had these data, we would be able to cost the private hospital admissions using actual costs for Barwon Health hospital episodes. We have not included the costs of non-admitted ED attendances to Barwon Health. Where patients were admitted, these costs are included in the admitted episode costs; however, patient-level cost data for non-admitted ED patients were not available. If non-admitted ED attendances mirror the admitted patient episodes, then both utilisation and costs are likely to be higher in the control group. Further to support this assumption is that where patients are managed in their home environment, it is likely that presentation to the ED without ward admission is the more likely outcome of earlier management of disease exacerbation. Alternatively, staff monitoring patients remotely may have encouraged presentation to the ED where symptoms suggested urgency.

## Conclusion

This evaluation indicates that telehealth care could be cost-effective when the program covers a large population. The difference and cost savings in hospitalisation is due to both a reduction in the number of acute admissions and a reduction in LOS in the intervention group. Although there are excellent telehealth monitoring programs in place, there are limited programs that have demonstrated cost-effectiveness analysis. We recommend that future telehealth interventions for chronic disease management evaluations include a cost-effectiveness analysis at immediate, mid-term and long-term time points and target a larger study sample to observe the real-life effect.
